# Changes in midlife fitness, body mass index, and smoking influence cancer incidence and mortality: A prospective cohort study in men

**DOI:** 10.1002/cam4.2383

**Published:** 2019-07-04

**Authors:** Trude E. Robsahm, Trond Heir, Leiv Sandvik, Erik Prestgaard, Steinar Tretli, Jan E. Erikssen, Ragnhild S. Falk

**Affiliations:** ^1^ Department of Research Cancer Registry of Norway Oslo Norway; ^2^ Oslo Ischemia Study Oslo University Hospital Oslo Norway; ^3^ Faculty of Medicine, Institute of Clinical Medicine University of Oslo Norway; ^4^ Oslo Centre for Biostatistics and Epidemiology Oslo University Hospital Oslo Norway; ^5^ Department of Cardiology Oslo University Hospital Oslo Norway

**Keywords:** body mass index, cancer, cardiorespiratory fitness, cohort study, midlife change, physical activity, smoke

## Abstract

Cancer prevention efforts include modification of unhealthy lifestyle, such as smoking cessation and resisting gain in body weight. Although physical activity is inversely related to risk of several cancers, it is poorly studied whether changes in physical activity or fitness influence future cancer risk. Thus, we aimed to investigate whether changes in midlife cardiorespiratory fitness (CRF), body mass index (BMI), and smoking habits influence cancer incidence and mortality. The study cohort includes 1689 initially healthy men, aged 40‐59 years. Measurements of CRF, BMI and information on smoking habits were collected in two repeated waves, 7 years apart. Cox regression models estimated associations as hazard rates (HR) with 95% confidence intervals (CI), between midlife changes in the modifiable lifestyle factors and cancer incidence and mortality. The men were followed prospectively for more than 30 years. Compared to CRF loss (>5%), improved CRF (>5%) was associated with lower cancer incidence (HR 0.81, 95% CI 0.67‐0.98) and mortality (HR 0.70, 95% CI 0.54‐0.92), and maintaining the CRF stable yielded lower cancer incidence (HR 0.76, 95% CI 0.61‐0.95). No association was seen for BMI gain, but maintaining the BMI stable was related to lower cancer incidence (HR 0.77, 95% CI 0.60‐0.98), compared to BMI loss. Continue smoking was associated with higher cancer incidence and mortality, compared to men who stopped smoking. In particular, this study adds new knowledge about the potential preventive role of CRF in cancer development and emphasizes lifestyle modification as a highly important effort in cancer prevention.

## 1 INTRODUCTION

In 2015, 17.5 million new cancer cases were diagnosed worldwide and 8.7 million cancer deaths occurred.^1^ The numbers are expected to increase considerably over the next decade due to population aging, population growth, and unhealthy lifestyle behaviors.^1^, ^2^ Tobacco smoking is a leading cause of cancer, related to 14 cancer sites,^2^, ^3^ while excess body fat, measured by body mass index (BMI), is related to 13 cancer sites.^3^, ^4^ However, cancer risk is shown to decrease after successful smoking cessation^2^ and after intentional loss in body fat.^2^, ^5^ Thus, primary cancer prevention efforts including modification of unhealthy lifestyle factors have become an urgent health issue.

About 20% of all cancer cases are estimated to be attributable to insufficient physical activity.^6^ Recently, the role of physical activity in cancer prevention was re‐reviewed by the Continuous Update Project, managed by the World Cancer Research Fund (WCRF)/American Institute for Cancer Research (AICR)^7^ and a large pooled analysis has emphasized an inverse relationship for 13 cancers.^8^ Whether changes in physical activity over time influence cancer incidence or mortality is barely studied.^9^ Such studies will increase our knowledge about the role of physical activity as a tool in prevention of cancer diseases.

Most studies on associations between cancer and physical activity are based on self‐reported information, which raises questions about validity of physical activity measures.^10^, ^11^ Physical fitness, a set of physiological attributes that are enhanced through regular physical activity, is less prone to misclassification and can capture health consequences of an active vs sedentary lifestyle. Most commonly used in health studies is cardiorespiratory fitness (CRF) that reflects aerobic activity, performed over time.^12^ To our knowledge, only one study has investigated the association between CRF changes and cancer (mortality only), reporting lower cancer mortality by increasing CRF,^13^ but none studying cancer incidence.

Based on a cohort of initially healthy men, aged 40‐59 years, with repeated measurements of modifiable lifestyle factors, we aimed to investigate the association between changes in CRF, BMI and smoking habits and cancer incidence and mortality.

## 2 METHODS

The Oslo‐Ischemia‐study is a comprehensive health survey established in 1972 aimed to examine the prevalence and development of coronary heart disease and other cardiovascular diseases in an initially healthy, employed male population.^14^, ^15^ In total, 2341 men in the age group 40‐59 years were invited, of whom 2014 (86%) provided informed consent in accordance with the Declaration of Helsinki, and completed the study protocol. A thorough screening of health records and a medical examination defined the men's health status. Inclusion into the study required absence of chronic disease, including malignant diagnosis within the last 5 years, and no medication use that would affect the ability to undertake a CRF test.^15^, ^16^, ^17^ After 12 hours of fasting and 8 hours of nonsmoking, a comprehensive clinical examination was conducted, including measurements of height, weight, blood pressure, and a panel of blood tests.^18^ CRF was assessed by a maximal exercise tolerance bicycle test, starting at 100 watts and incremented by 50 watts every 6 minutes.^15^ Participants were encouraged to continue exercising until exhaustion. Information about smoking habits was collected by questionnaire. A new wave of data collection was performed after 7 years, using the same examinations, procedures, and questionnaire.^19^

The Oslo‐Ischemia‐study was linked to the Cancer Registry of Norway and the Norwegian Cause of Death Registry, for information on cancer diagnoses, vital status, and death throughout 2012, using the personal identification number assigned to all Norwegian citizens. The Cancer Registry of Norway has registered data on all cancers diagnosed in the Norwegian population since 1953. Mandatory reporting from several independent sources ensures completeness and high data quality.^20^, ^21^ Information on cancer is based on the 10th edition of the World Health Organization's (WHO) International Classification of Diseases (ICD‐10) codes for cancer.

The Cause of Death Registry holds information on all deaths among Norwegian citizens. A physician determines the cause of death (ICD‐10 codes) and the reporting is mandatory by law. The underlying cause of death was used to identify deaths from cancer.

**Figure 1:**
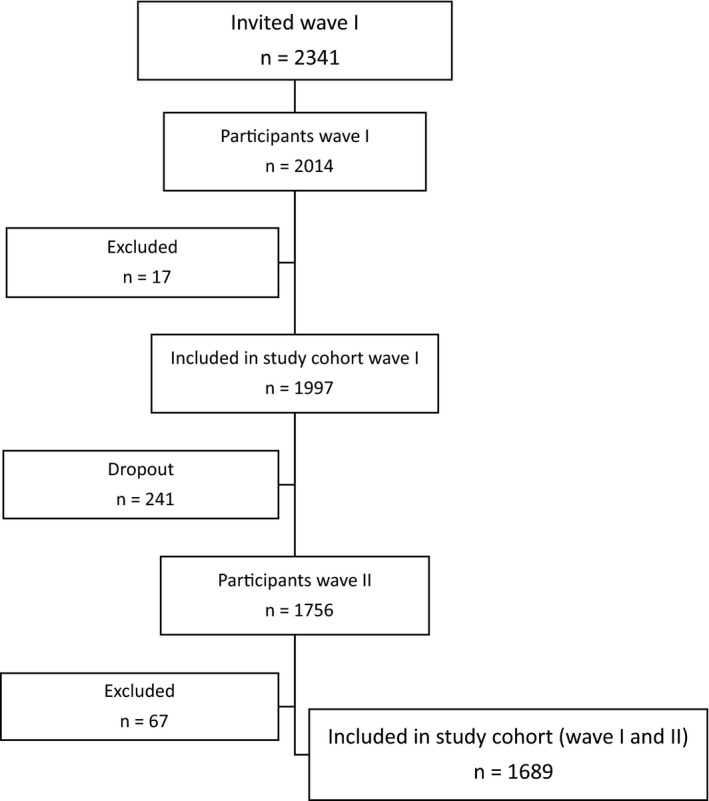
In total, 2341 employed men (40‐59 years of age) were invited to the health survey, wave I, of whom 2014 completed the study protocol. Exclusion from wave I was due to history of cancer (n = 15) and missing data on vital status (n = 2), leaving 1997 men in the study cohort. In wave II, 241 men were not available due to death, illness, and long travel distance (dropout), and 67 men were excluded due to cancer between the waves, leaving 1689 men in the study cohort

Of the initial 2014 men, 17 were excluded, either due to a cancer diagnosis prior to the first wave of data collection (n = 15) or due to missing data on vital status (n = 2), leaving 1997 men in the study cohort. In the second wave, 1756 (88%) of the men participated. Main reasons for not participating were death, illness that made it impossible to undertake the CRF test, and long travel distance. Selection and implementation procedures have previously been reported in detail.^16^, ^19^ Furthermore, 67 men were excluded due to a cancer diagnosis between the two data collection waves, leaving 1689 presumably healthy men for statistical analysis (Figure 1).

Permission to link the data was provided by the Regional Committees for Medical and Health Research Ethics.

### 2.1 Exposure variables

Age at inclusion was divided into four groups (<45, 45‐49, 50‐54, 55 + years). In each wave, CRF was measured as total work (sum of work performed in the bicycle test) divided by body weight, kilopond meter (kpm)/kg. Based on objectively measured height and weight, individual BMI was calculated as kg/m^2^ and, according to the categorization by WHO,^22^ classified into low/normal weight (BMI < 25 kg/m^2^) and overweight/obese (BMI ≥ 25 kg/m^2^). Based on self‐reported information (questionnaire) about smoking, the men were categorized as never, former or present smokers.

### 2.2 Changes in exposure variables

The mean time interval between the data collection waves was 7.3 years (SD 0.76). CRF values from both waves were standardized to age 50 and 57, respectively, to account for decreasing fitness by increasing age, due to a decline in physical activity and muscle mass.^23^ The individual absolute change per year was calculated by dividing the age‐standardized CRF change by the number of years between the waves. The age‐standardized relative change in CRF was calculated as the difference between the age‐standardized CRF in wave II minus age‐standardized CRF in wave I, divided by age‐standardized CRF in wave I. To ensure the groups with CRF changes to be large enough, change in age‐standardized CRF was defined by: >5% increase (more fit), >5% decrease(less fit), and ≤5% changes (stable).

Similarly, we calculated the absolute change in BMI per year and the relative change in BMI based on BMI measurements in the two data collection waves. Due to minor changes in BMI over the 7‐year time‐period, relative change in BMI was defined by: >5% increase or decrease was categorized as “gain” or “loss”, respectively, while changes ≤5% were categorized as “stable”.

Based on self‐reported information on smoking in the two waves, the men were categorized into three groups of smoking change: (a) never‐smoker, (b) cessation (ie smoking cessation before wave I or between the waves), (c) smoker (ie present smoker in both waves or in wave II only).

### 2.3 Statistical methods

The study cohort was followed from the date of wave II to the date of cancer diagnosis, date of death, emigration or end of follow‐up, at 31 December 2012, whichever occurred first.

Descriptive analyses were conducted for characteristics at each wave and presented as means with standard deviations (SD), range and percentages (%). Cox regression models were used to evaluate the etiological relationship between midlife changes in the modifiable lifestyle factors (CRF, BMI and smoking) and cancer incidence and mortality, respectively. For cancer incidence and mortality, time to event was calculated as time from date of wave II to date of primary cancer diagnosis or to date of death from cancer, respectively, otherwise considered as censored. In the final Cox models, age, CRF, BMI, and smoking (all in wave I) were included as independent variables.

Effect estimates were presented as hazard ratios (HRs) with 95% confidence intervals (CI). Analyses of Schoenfeld residuals verified that the proportional‐hazards assumption was met. To eliminate the possibility that a low CRF score resulted from an ongoing cancer disease (reverse causality), additional analyses were conducted, restricted to men still alive and cancer free 10 years after start of follow‐up. Lastly, a Kaplan‐Meier plot was made to illustrate cumulative cancer incidence and mortality throughout the follow‐up time (Figure S1).

All statistical analyses were performed using Stata 14 (StataCorp, College Station, TX). The statistical significance level was set at 5%.

## 3 RESULTS

Characteristics of the men are presented separately for the two waves of data collection (Table 1). The mean age was 49.3 and 56.8 years, respectively. The mean CRF decreased between the data collection waves, representing an absolute age‐standardized reduction of 2.13 kpm/kg per year (SD 5.7). With regard to age‐standardized relative change in CRF, 58.6% became less fit (>5% reduction), 23.9% became more fit (>5% increase) and 17.5% remained stable (≤5% change). The mean body weight increased with less than 1 kilo between the data collection waves. In general, men were of normal weight and the mean BMI changed insignificantly, from 24.6 to 24.8 kg/m^2^. The proportion of smokers was about 15% lower in wave II, compared to wave I.

**Table 1:** Characteristics of the study cohort at the two waves of data collection, on average 7.3 years (SD 0.76) apart

Characteristics	Wave I n = 1997	Wave II^a^ n = 1689
Age [y], mean (SD; range)	49.3 (5.5; 37.0‐62.0)	56.8 (5.5; 44.4,69.0)
CRF [kpm/kg], mean (SD; range)	146.2 (57.5; 21.1‐553.8)	134.5 (62.9; 8.0,542.7)
CRF [kpm/kg], age‐standardized absolute change per year, mean (SD; range)		−2.13 (5.7; −61.7,51.6)
CRF [kpm/kg], age‐standardized relative change		
Less fit [> −5%], n (%)		990 (58.6)
Stable [± 5%], n (%)		296 (17.5)
More fit [> +5%], n (%)		403 (23.9)
Weight [kg], mean (SD; range)	76.8 (9.9; 50.0‐122.5)	77.6 (10.0; 52.0‐124.0)
Height^b^ [cm], mean (SD; range)	176.9 (6.2; 153.5‐198.5)	176.9 (6.2; 153.5‐198.5)
BMI [kg/m^b^], mean (SD; range)	24.6 (2.8; 17.2‐38.9)	24.8 (2.8; 16.9‐38.7)
<25, n (%)	1210 (60.6)	972 (57.6)
≥25, n (%)	787 (39.4)	717 (42.4)
BMI [kg/m^b^] absolute change per year, mean (SD; range)		0.03 (0.19; −0.96‐1.12)
BMI [kg/m^b^] relative change		
Loss [>−5%], n (%)		195 (11.5)
Stable [± 5%], n (%)		1165 (70.0)
Gain [> +5%], n (%)		329 (19.5)
Smoking		
Never, n (%)	502 (25.1)	415 (24.6)
Former, n (%)	620 (31.1)	782 (46.3)
Present, n (%)	875 (43.8)	492 (29.1)
Smoking change^c^		
Never‐smoker, n (%)		415 (24.6)
Cessation, n (%)		782 (46.3)
Smoker, n (%)		492 (29.1)

SD, standard deviation; CRF, cardiorespiratory fitness [Kilopond meter, kpm/kg]; BMI, body mass index (weight/height).

a Include all men with cardiorespiratory fitness (CRF) measured in both data collection waves and no cancer diagnosis prior to wave II

b Measured in wave I only

c Never‐smoker (never), Cessation (cessation before wave I or between wave I and II), smoker (present in both waves or wave II)

Overall, 638 incident cancer cases and 352 cancer deaths occurred over an average of 20.5 years (SD 9.2, range 0.1‐33.8) since start of follow‐up (wave II). Table S1 shows the number of cancer cases by site and according to ICD‐10 codes. Table 2 presents HRs of overall cancer incidence by midlife changes in CRF, BMI, and smoking habits from crude analyses (model I), when adjusted for the respective modifying lifestyle variables (model II) and from fully adjusted models (model III). A slightly and statistically significant lower cancer incidence was found by annual absolute increase in age‐standardized CRF. Compared to men who became “less fit”, men with a stable CRF and men who became “more fit” had significantly lower cancer incidence with HRs of 0.76 (95% CI 0.61‐0.95) and 0.81 (95% CI 0.67‐0.98), respectively. An annual absolute increase in BMI was not associated with cancer incidence. When compared to BMI loss, maintaining a stable BMI was associated with lower incidence (HR 0.77, 95% CI 0.68‐0.98). Compared to never‐smokers, the cancer incidence was significantly higher among persistent smokers (1.46, 95% CI 1.19‐1.80). For the cessation group, the HR (0.99, 95% CI 0.82‐1.20) was similar to that of never‐smokers.

**Table 2:** Hazard ratio (HR) with 95% confidence interval (CI) for cancer incidence by change in cardiorespiratory fitness (CRF), body mass index (BMI), and smoking habits, in initially healthy men, based on two waves of data collection on average 7.3 years (SD 0.76) apart

		Cancer incidence		
	n*/cases*	Model I^a^ HR (95% CI)	Model II HR (95% CI)	Model III HR (95% CI)
CRF, age‐standardized absolute change per year (cont.)		0.98 (0.97,0.99)	0.98 (0.97,0.99) b	0.98 (0.96,0.99) c
CRF, age‐standardized relative change				
Less fit (> 5%)	*915/338*	1.00	1.00^b^	1.00^c^
Stable (± 5%)	*404/150*	0.75 (0.60,0.93)	0.76 (0.61,0.95)^b^	0.76 (0.61,0.95)^c^
More fit (> 5%)	*370/150*	0.80 (0.67,0.96)	0.82 (0.68,0.99)^b^	0.81 (0.67,0.98)^c^
BMI, absolute change per year (cont.)		0.85 (0.56,1.30)	0.83 (0.54,1.27)^d^	0.91 (0.59,1.41)^e^
BMI, relative change				
Loss (> 5%)	*197/79*	1.00	1.00^d^	1.00^e^
Stable (± 5%)	*1160/434*	0.75 (0.59,0.95)	0.75 (0.59,0.96)^d^	0.77 (0.60,0.98)^e^
Gain (>5%)	*332/125*	0.82 (0.62,1.08)	0.82 (0.61,1.09)^d^	0.85 (0.64,1.15) e
Smoking change^h^				
1 Never‐smoker	*415/158*	1.00	1.00^f^	1.00^g^
2 Cessation	*782/285*	0.99 (0.82,1.20)	0.99 (0.81,1.20) f	0.99 (0.82,1.20)^g^
3 Smoker	*492/195*	1.41 (1.14,1.74)	1.42 (1.14,1.76)^f^	1.46 (1.19,1.80)^g^

a Crude analysis.

b Adjusted for smoke and BMI in wave I.

c Additionally adjusted for CRF in wave I.

d Adjusted for smoke and CRF at in wave I.

e Additionally adjusted for age and BMI in wave I.

f Adjusted for CRF and BMI in wave I.

g Additionally adjusted for age in wave I.

h Never‐‐smoker (never), cessation (cessation before wave I or between wave I and II), smoker (present in both waves or wave II only).

Table 3 presents the associations between midlife changes in the modifiable lifestyle factors and cancer mortality. An annual absolute increase in age‐standardized CRF was associated with lower cancer mortality (HR 0.96, 95% CI 0.94‐0.99). Compared to men who became less fit, those who became more fit had lower cancer mortality (HR 0.70, 95% CI 0.54‐0.92), while no significant difference was seen for men who maintained the age‐adjusted CRF stable. No differences in cancer mortality were seen between the BMI categories. Smokers had significantly higher cancer mortality (HR 1.75, 95% CI 1.32‐2.31), while HR for the cessation group (0.87 95% CI 0.67‐1.15) was not different from that of never‐smokers.

**Table 3:** Hazard ratio (HR), 95% confidence interval (CI) for cancer mortality by change in cardiorespiratory fitness (CRF), body mass index (BMI) and smoking habits, in initially healthy men, based on two waves of data collection on average 7.3 years (SD 0.76) apart

		Cancer mortality		
	n*/deaths*	Model I^a^ HR (95% CI)	Model II HR (95% CI)	Model III HR (95% CI)
CRF, age‐standardized absolute change per year (cont.)		0.97 (0.95,0.99)	0.97 (0.95,0.99)^b^	0.96 (0.94,0.99)^c^
CRF, age‐standardized relative change				
Less fit > 5%	*915/191*	1.00	1.00^b^	1.00^c^
Stable ± 5%	*404/91*	0.83 (0.62,1.10)	0.84 (0.64,1.12)^b^	0.83 (0.62,1.11)^c^
More fit > 5%	*370/70*	0.71 (0.55,0.92)	0.74 (0.57,0.96)^b^	0.70 (0.54,0.92)^c^
BMI, absolute change per year (cont.)		0.81 (0.46,1.44)	0.78 (0.44,1.36)^d^	1.03 (0.60,1.84)^e^
BMI, relative change > 5%				
Loss > 5%	*197/41*	1.00	1.00^d^	1.00^e^
Stable ± 5%	*1160/245*	0.83 (0.60,1.17)	0.87 (0.62,1.21)^d^	0.93 (0.66,1.20)^e^
Gain > 5%	*332/66*	0.85 (0.58,1.26)	0.84 (0.57,1.24)^d^	0.99 (0.66,1.48)^e^
Smoking change^h^				
1 Never‐smoker	*415/86*	1.00	1.00^f^	1.00^g^
2 Cessation	*782/139*	0.87 (0.66,1.14)	0.87 (0.66,1.13)^f^	0.87 (0.67,1.15)^g^
3 Smoker	*492/127*	1.72 (1.31,2.26)	1.67 (1.27,2.20)^f^	1.75 (1.32,2.31)^g^

a Crude analysis

b Adjusted for smoke and BMI in wave I

c Additionally adjusted for fitness in wave I

d Adjusted for smoke and fitness in wave I;

e Additionally adjusted for age and BMI in wave I

f Adjusted for fitness and BMI in wave I

g Additionally adjusted for age in wave I.

h Never‐smoker (never/), cessation (cessation before wave I or between wave I and II), smoker (present in both waves or wave II only)

Excluding the first 10 years of follow‐up reduced the estimates for changes in CRF and BMI, and except for stable vs lower BMI, the estimates were not statistically significant (Table S2).

## 4 DISCUSSION

The main finding in this long‐term prospective cohort study was the lower cancer incidence (19%) and mortality (30%) found in men who became more fit, when compared to men who became less fit. Lower cancer incidence was also found for men who maintained a stable CRF (24%) and those who maintained a stable BMI (23%), while no association was found for cancer mortality. To continue smoking was associated with the highest cancer incidence and mortality, while smoking cessation gave similar risk estimates as found for never‐smokers.

The relationship between physical activity and cancer risk has been thoroughly examined and it is well established that physical activity has beneficial effect. Recently, the WCRF expert report from the Continuous Update Project re‐reviewed the research globally. The report concludes with strong evidence, that being physically active decreases the risk of colon, breast, and endometrial cancer and, with weaker evidence for cancers of esophagus, lung, and liver.^7^ A large pooled analysis shows that physical activity was inversely related to risk of 13 cancer types.^8^ Results from the Copenhagen Male Study, including healthy middle‐aged men, showed that CRF was inversely related both to cancer incidence and mortality, after more than 40 years of follow‐up.^24^, ^25^ Moreover, long‐term effects of changes in physical activity and measured CRF has been demonstrated for cardiovascular diseases and overall survival.^26^ To our knowledge, whether such changes modify subsequent cancer incidence and mortality has been examined in two studies only. One cohort study, including both sexes, found no association between changes in physical activity, over a 15‐year period, and colon cancer, for neither incidence nor mortality.^9^ The study was based on self‐reported physical activity that could have introduced misclassification, and thus leads to underestimation of the effect. The other study examined associations between changes in CRF in middle‐aged men, over a 6‐year period, and overall cancer mortality.^13^ A stable or increased CRF was associated with lower cancer mortality. Our results were in line with this finding and support previous studies based on the *Oslo‐Ischemia‐study*, demonstrating that changes in CRF increase longevity.^19^ Adjustments of potential confounders and levels of the lifestyle variables in wave I did not affect our results. On average, the men became less fit, over the 7‐year period, while about 40% maintained or increased their CRF level. After accomplishing wave I, the participants received recommendations for health improvement,^19^ which might have led to improved CRF in the following wave. Our findings indicate that changes in midlife CRF impact on future cancer risk and emphasize the beneficial role of physical activity in cancer prevention.

Evidence links obesity to incidence and mortality of several cancer types.^3^, ^27^ Moreover, BMI gain in adulthood, assumed to reflect fatness as it mostly results from accumulation of fat rather than of lean tissue, is positively related to the risk of breast and endometrial cancer and probably also related to pancreatic and skin cancer.^27^, ^28^ Compared to BMI loss, we found no association between BMI gain and cancer incidence. About 60% of the cohort were within the range of normal weight (BMI < 25), in both data collection waves. The limited increase in BMI, over the 7‐year time span may result from recommendations for health improvements given after the first data collection wave.^19^ The health advice may also have led to smoking cessation, which is often followed by weight gain.^29^ In the second wave, the proportion of smokers was reduced by 15% and descriptive analysis revealed that men who stopped smoking had the highest weight gain. Thus, a reasonable explanation of not observing a relationship between BMI gain and cancer risk may be due to the beneficial health consequences of smoking cessation, which is more important than the potential disadvantages of BMI gain. On the other hand, the highest cancer incidence was seen in men with loss in BMI, which can have resulted from ongoing cancer development.^30^ However, sensitivity analysis, excluding the first 10 years of follow‐up to minimize the risk of reverse causation, did not change this result. Maintaining a stable BMI was associated with lower cancer incidence, which is in agreement with the current advice from WCRF/AICR.^29^ No association was found between BMI changes and cancer mortality, in line with most previous studies,^13^, ^31^, ^32^ although one study observed a positive relationship for prostate cancer.^33^

Present smokers had the highest cancer incidence and mortality. We found no significant differences, for neither cancer incidence nor mortality, between never‐smokers and men who stopped smoking, in line with the current evidence that shows how successful smoking cessation significantly drops cancer risk.^2^

The main strength of the present study is the use of objective measured CRF, height, and weight, and it is one of the first to use repeated measurements of CRF to study associations with cancer outcomes. The CRF score is a reliable measure of aerobic activity over time, although we are aware that a CRF test does not capture all kind of activity (ie light activity that does not affect the aerobic capacity). From the Cancer Registry of Norway we have complete and valid information on cancer diagnoses and cause of death during this more than three decades of follow‐up. The cohort was found to be representative for their age group of men in the given time‐period, regarding cancer incidence in the region they were recruited from.^17^ The study also has limitations that need to be considered when interpreting the results. First, for inclusion into the study the men had to participate in both data collection waves, requiring survival to wave II, which may have selected a healthier group of persons. Second, for inclusion into wave I, the men were required to be healthy, but whether a low‐score CRF test could result from an undiagnosed cancer disease could not be ruled out. We conducted sensitivity analyses, excluding the first 10 years after the start of follow‐up showing that the statistical significance for the association between CRF change and cancer incidence disappeared. Third, the inclusion of men only makes the results less generalizable, in particular for BMI, as several common female cancers are related to BMI (eg breast and endometrial cancer). Fourth, information on dietary factors and alcohol intake was lacking, factors that may affect risk of gastrointestinal cancers in particular. Neither were we able to consider socioeconomic factors (ie education, income), which is a limitation as health concern varies between socioeconomic levels. However, Norway has a public health care system financed by taxation and founded on the principles of universal access and care. Lastly, information on pack‐years was not given and, thus, residual confounding of smoking cannot be excluded.

In conclusion, this prospective long‐term follow‐up study of initially healthy men reveals advantages of changes in midlife to a healthier lifestyle, both for cancer incidence and mortality. In particular, this study adds new knowledge about risk reductions associated with a stable or improved CRF. Avoidance of weight gain might reduce cancer incidence and the results support that smoking cessation reduce cancer incidence and mortality. The results emphasize lifestyle modification as a highly important effort for cancer prevention.

## CONFLICT OF INTEREST

The authors declare no competing interests.
