# Supplementary material for: Changes in midlife fitness, body mass index, and smoking influence cancer incidence and mortality: A prospective cohort study in men
Source: Cancer Med. 2019 Jul 4;8(10):4875–82. doi: 10.1002/cam4.2383 (PMC6712445; doi:10.1002/cam4.2383)
Supplement: Supplementary file 2 [file CAM4-8-4875-s002.docx]

**Supplementary tables**

**Table S1** Number of cancer cases by cancer location and according to the International Classification of Diseases (ICD-10) codes for cancer

| **Cancer location** | **ICD-10** |  | **Number of cancer cases** |
| --- | --- | --- | --- |
| Head and neck | C00-C14 |  | 15 |
| Esophagus | C15 |  | 9 |
| Stomach | C16 |  | 20 |
| Colon | C18, C19 |  | 61 |
| Rectum | C20 |  | 34 |
| Liver, Gallbladder | C22, C23 |  | 6 |
| Pancreas | C25 |  | 18 |
| Lung | C34 |  | 68 |
| Prostate | C61 |  | 172 |
| Kidney, urinary tract | C64, C66 |  | 21 |
| Bladder | C67 |  | 54 |
| Skin, Cutaneous melanoma | C43 |  | 35 |
| Skin, Squamous cell carcinoma | C44 |  | 29 |
| Central nervous system | C70-C72 |  | 18 |
| Lymphoma | C81-C85 |  | 22 |
| Leukemia | C91-C95 |  | 27 |
| Other cancers* | C76, C32, C38, C49, C62, C69, C73-C75 |  | 28 |

* Other cancers includes larynx, heart/plaura, connecting tissue, testis, eye, endocrine organs and not further specified

**Table S2** Hazard ratios (HRs) for cancer incidence and mortality, with 95% confidence intervals (95% CIs), by midlife change in cardiorespiratory fitness (CRF), body mass index (BMI) and smoking habits, when excluding the first 10 years of follow-up

|  | **Cancer incidence**  *(n=1426/504)*  HR (95% CI) | **Cancer mortality**  *(n=1426/264)*  HR (95% CI) |
| --- | --- | --- |
| CRF, age-standardized absolute change per year^1^ | 0.98 (0.97,1.00) | 0.97 (0.95,0.99) |
| CRF, age-standardized relative change^1^  Less fit > 5%  Stable ± 5%  More fit >5 % | 1.00  0.80 (0.62,1.02)  0.87 (0.71,1.08) | 1.00  0.88 (0.64,1.22)  0.78 (0.58,1.05) |
| BMI, absolute change per year^2^ | 1.02 (0.63,1.68) | 1.32 (0.68,2.54) |
| BMI, relative change >5%^2^  Loss > 5%  Stable ± 5%  Gain > 5% | 1.00  0.73 (0.55,0.97)  0.91 (0.66,1.25) | 1.00  0.98 (0.65,1.48)  1.16 (0.73,1.87) |
| Smoking change^3^  1 Never-smoker^4^  2 Cessation^5^  3 Smoker^6^ | 1.00  0.98 (0.79,1.21)  1.41 (1.11,1.79) | 1.00  0.78 (0.57,1.05)  1.64 (1.19,2.25) |

^1^adjusted for fitness, BMI and smoke in wave I; ^2^adjusted for age, fitness, BMI and smoke in wave I; ^3^adjusted for age fitness and BMI in wave I; ^4^Never-smoker (never); ^5^cessation (cessation before wave I or between the waves); ^6^smoker (present in both waves or wave II only)
